# New parameters of the 8th edition AJCC/UICC T category in nasopharyngeal carcinoma: Cervical vertebrae invasion and parotid gland invasion

**DOI:** 10.1002/ctm2.202

**Published:** 2020-11-10

**Authors:** Rui Guo, Shun‐Xin Wang, Yong Hu, Shao‐Bo Liang, Ling‐Long Tang, Li‐Zhi Liu, Li Tian, Xiao‐Tong Luo, Jun Ma, Yan‐Ping Mao

**Affiliations:** ^1^ Department of Radiation Oncology Sun Yat‐sen University Cancer Center, State Key Laboratory of Oncology in South China, Collaborative Innovation Center for Cancer Medicine, Guangdong Key Laboratory of Nasopharyngeal Carcinoma Diagnosis and Therapy Guangzhou China; ^2^ Department of Radiation Oncology Hanzhong Central Hospital Hanzhong China; ^3^ Radiotherapy Department of Nasopharyngeal Carcinoma First People's Hospital of Fo Shan Affiliated to Sun Yat‐sen University Foshan China; ^4^ Imaging Diagnosis and Interventional Center Sun Yat‐sen University Cancer Center, State Key Laboratory of Oncology in South China, Collaborative Innovation Center for Cancer Medicine, Guangdong Key Laboratory of Nasopharyngeal Carcinoma Diagnosis and Therapy Guangzhou China; ^5^ State Key Laboratory of Biocontrol, School of Life Sciences Sun Yat‐sen University Guangzhou China

Dear Editor,

The anatomic extent or stage of cancer provides the critical benchmarks and standards for defining prognosis and for determining the best treatment approach, and it also presents stratification criteria for clinical trials and consistent nomenclature for exchanging experience.^1^, ^2^ The AJCC/UICC TNM staging system is widely used in tumor staging worldwide due to its periodic revision supported by high‐quality evidence.^1^, ^2^ In response to the better delineation of tumor extent resulting from advances in imaging techniques, the latest released 8th edition staging system of nasopharyngeal carcinoma (NPC) empirically introduces cervical vertebrae invasion (CVI) into T3 and parotid gland invasion (PGI) into T4. However, data to support this experience‐based modification remain unavailable. In order to provide evidence‐based data for future management, we performed a large‐scale magnetic resonance imaging (MRI)‐based study to investigate the characteristics, treatment, and prognosis of CVI and PGI. Furthermore, we excluded CVI and PGI from the frame of T category, and named the remaining frame of T category as T1′, T2′, T3′, and T4′ to evaluate the roles of the two parameters in the 8th edition T category of NPC.

This retrospective analysis enrolled 2190 patients with newly diagnosed, pathologically proven, nonmetastatic NPC who underwent intensity‐modulated radiotherapy‐based treatment. The key raw data have been uploaded onto the Research Data Deposit public platform (http://www.researchdata.org.cn), with the approval RDD number as RDDA2020001728. For the whole series, the incidence of CVI (Figure S1) and PGI (Figure S2) was 3.6% (79/2190) and 2.1% (45/2190), respectively. Patient characteristics categorized by CVI and PGI are detailed in Table S1. A higher proportion of patients with CVI/PGI had advanced T′ disease (T3′ and T4′) and overall stage (III′ and IVA′), and received induction chemotherapy than those without CVI/PGI (all *P *< .05). In addition, patients with CVI or PGI were found to have extremely extensive local tumor extension, with 100% and 100% showing parapharyngeal space invasion, 98.7% and 97.8% showing adjacent soft tissue invasion, 100% and 97.8% showing skull base or paranasal sinus invasion, and 54.4% and 57.8% showing invasion of other T4 parameters, respectively (Table S2). CVI and PGI were both associated with invasion into oropharynx, parapharyngeal space, adjacent soft tissues (medial/lateral pterygoid muscle and prevertebral muscle), pterygoid process, basis of sphenoid bone, clivus, petrous apex, great wing of sphenoid bone, foramen magnum, skull base foramina, paranasal sinus, intracranial, orbit, and extensive soft tissues in univariable analysis (all *P *< .05). Only oropharynx and foramen magnum invasion remained significantly correlated with CVI in multivariable logistics analysis (all *P *< .05), whereas oropharynx, foramen magnum, medial pterygoid muscle, lateral pterygoid muscle, and clivus invasion were independently associated with PGI (all *P *< .05) (Table S2).

Radiation dosimetry for the 94 patients with CVI and/or PGI is summarized in Table S3. According to the recommended maximum acceptance criteria in radiation therapy planning for NPC,^3^ 40 of 94 (42.6%) patients had inadequate tumor coverage (*D*_min_ of primary gross tumor volume < 66.5 Gy); 25 (26.6%) and 73 (77.7%) underwent excessive radiation to spinal cord (*D*_0.03cc_ planning risk volume [PRV] > 50 Gy) and brainstem (*D*_0.03cc_ PRV > 60 Gy), respectively. During follow‐up, one of the patients with inadequate tumor coverage developed local failure, which was considered an in‐field failure for its location within the applicable 95% isodose line; of the patients who underwent an overdose of radiation, two showed radiation‐induced brainstem damage on MRI, and none suffered from radiation damage to spinal cord. Overall, it is challenging to achieve the optimal balance between local control and potentially serious late complications when planning for patients with CVI/PGI.

Median follow‐up was 94.9 (4.0‐118.7) months for the whole cohort; the 5‐year overall survival (OS), disease‐free survival (DFS), distant metastasis‐free survival (DMFS), and local relapse‐free survival (LRFS) rates were 62.0% and 86.1% (*P *< .001), 58.2% and 78.6% (*P *< .001), 67.8% and 87.5% (*P *< .001), and 89.7% and 93.6% (*P *= .082) for patients with and without CVI, respectively; the corresponding rates for patients with and without PGI were 64.4% and 85.6% (*P *< .001), 60.0% and 78.2% (*P *= .001), 67.2% and 87.2% (*P *< .001), and 92.1% and 93.5% (*P *= .986), respectively (Figure 1). Multivariable Cox analysis identified CVI as an independent adverse indicator of OS (hazard ratio [HR] = 1.65; *P *= .006), DFS (HR = 1.41; *P *= .046), and DMFS (HR = 1.66; *P *= .020), whereas PGI showed no significance for any endpoint (Table S4).

**FIGURE 1:**
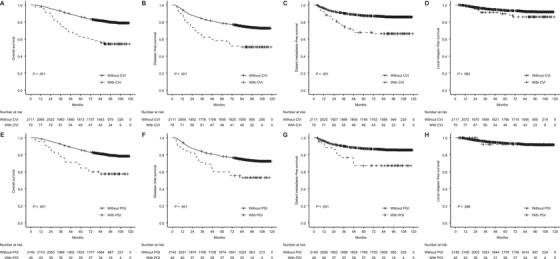
Kaplan‐Meier curves of OS, DMFS, LRFS, and DFS for patients without and with CVI (A, B, C, and D) and patients without and with PGI (E, F, G, and H) Abbreviations: CVI, cervical vertebrae invasion; DFS, disease‐free survival; DMFS, distant metastasis‐free survival; LRFS, local relapse‐free survival; OS, overall survival; PGI, parotid gland invasion.

The survival rates between T2′ and T3′ were extremely similar in this series, with the 5‐year OS, DFS, DMFS, and LRFS being 86.9% and 85.7% (*P *= .283), 79.4% and 78.0% (*P *= .450), 88.5% and 87.1% (*P *= .734), and 94.2% and 94.2% (*P *= .332), respectively (Figure S3). Thus, we grouped patients with T2′ and T3′ disease as a whole, leading to the three T′ category groups. The whole cohort was randomly assigned to a training set (n = 1095) and a validation set (n = 1095). In the training set, we incorporated CVI and PGI into the three T′ category groups by using a separate recursive partitioning analysis (RPA)^4^ (Figure 2). The 5‐year OS of patients in T2′‐3′ group with CVI and/or PGI ranged from 67% to 75%, which was worse than that of patients in T2′‐3′ group without CVI/PGI (86%), and comparable to that of patients in T4′ (77%). Consequently, the T category proposed by RPA defined both CVI and PGI as T4 disease. A comparison between the proposed and the 8th edition T category was carried out based on OS in the validation set. Compared to the 8th edition T category, the proposed T category showed better performance after 1000 bootstrap replicates^5^ with lower values of hazard consistency (0.652 vs 1.628), hazard discrimination (0.523 vs 0.794), and sample size balance (0.399 vs 0.448) and a higher value of outcome prediction^6^ (24.9 vs 24.8) (Table S5).

**FIGURE 2:**
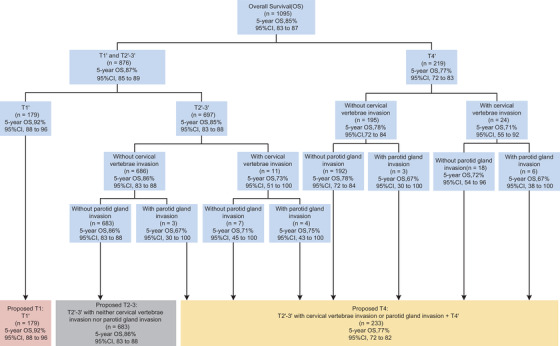
Recursive partitioning analysis incorporating parameters of cervical vertebrae invasion and parotid gland invasion into the three T′ category groups in the training set (n = 1095) Abbreviations: CI, confidence interval; OS, overall survival.

This study revealed CVI and PGI were rare and usually accompanied by extensive local extension in NPC. The presence of CVI/PGI was associated with advanced disease and poor prognosis, which served as evidence for the introduction of CVI and PGI into the 8th edition T category. CVI was identified as an independent negative indicator of OS, DFS, and DMFS in NPC, and its potential to be classified as T4 disease needs further validation.

## AUTHOR CONTRIBUTIONS

Rui Guo, Shun‐Xin Wang, Yong Hu, Jun Ma, and Yan‐Ping Mao conceptualized the study. Rui Guo, Yong Hu, Jun Ma, Shao‐Bo Liang, Ling‐Long Tang, and Yan‐Ping Mao designed the study. Rui Guo, Shun‐Xin Wang, Yong Hu, Shao‐Bo Liang, Li‐Zhi Liu, and Yan‐Ping Mao contributed acquired data. Rui Guo, Shun‐Xin Wang, Xiao‐Tong Luo, and Yan‐Ping Mao contributed with formal analysis. All authors prepared, reviewed, and edited the manuscript. All authors read and approved the final manuscript.

## FUNDING INFORMATION

This work was supported by the National Natural Science Foundation of China (81930072, 81672988, and 81803052); the Key‐Area Research and Development Program of Guangdong Province (2019B020230002); and the Sun Yat‐Sen University Clinical Research 5010 Program (2017‐FXY‐114).

## ETHICS APPROVAL AND CONSENT TO PARTICIPATE

This study was approved by the Institutional Ethical Review Board of Sun Yat‐sen University Cancer Center, and the requirement for written informed consent was waived.

## CONFLICT OF INTEREST

The authors declare no conflict of interest.

## Supporting information

Figure S1. The MR images of a 30‐year‐old man with invasion of cervical vertebrae presented in (A) axial T1‐weighted image, (B) axial T1‐weighted fat‐suppressed contrast‐enhanced image, (C) sagittal T1‐weighted image, (D) sagittal T1‐weighted contrast‐enhanced image, (E) coronal T1‐weighted image, and (F) coronal T1‐weighted fat‐suppressed contrast‐enhanced image: the mass of nasopharynx (solid thick arrow) posteriorly extended through the clivus (hollow thick arrow) and foramen magnum (solid triangle) and further invaded cervical vertebrae (solid arrowhead) and prepontine cistern (solid thin arrow).

Figure S2. The MR images of a 41‐year‐old woman with invasion of parotid gland presented in (A) axial T1‐weighted image, (B) axial T1‐weighted contrast‐enhanced image, (C) coronal T1‐weighted image, and (D) coronal T1‐weighted fat‐suppressed contrast‐enhanced image: the mass of nasopharynx (thick arrow) posterolaterally extended through the parapharyngeal fat space and adjacent soft tissue (thin arrow), and further invaded the deep lobe of parotid gland (arrowhead), resulting in a heterogeneous mass.

Figure S3. Kaplan‐Meier curves of overall survival (A), disease‐free survival (B), distant metastasis‐free survival (C), and local relapse‐free survival (D) according to the T’ category (in which cervical vertebrae invasion and parotid gland invasion were excluded from the frame of the 8^th^ edition T category) for the whole cohort.

Tables

## Data Availability

The datasets are available from the corresponding author upon reasonable request.
