# Supplementary figures and images for: New parameters of the 8th edition AJCC/UICC T category in nasopharyngeal carcinoma: Cervical vertebrae invasion and parotid gland invasion
Source: Clin Transl Med. 2020 Nov 10;10(7):e202. doi: 10.1002/ctm2.202 (PMC7654628; doi:10.1002/ctm2.202)

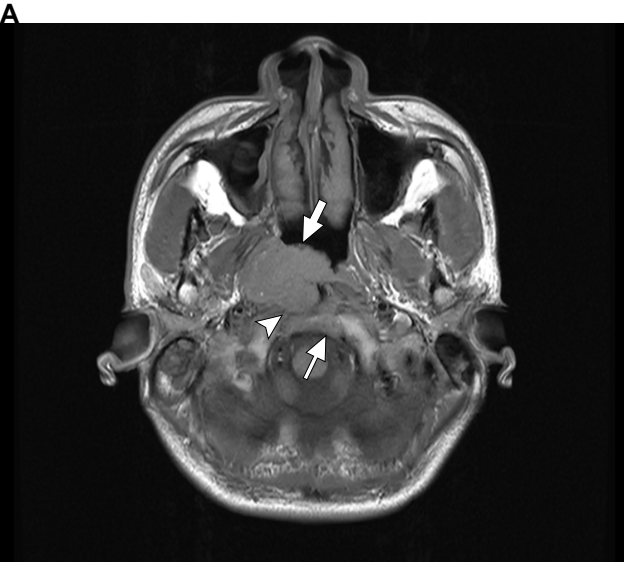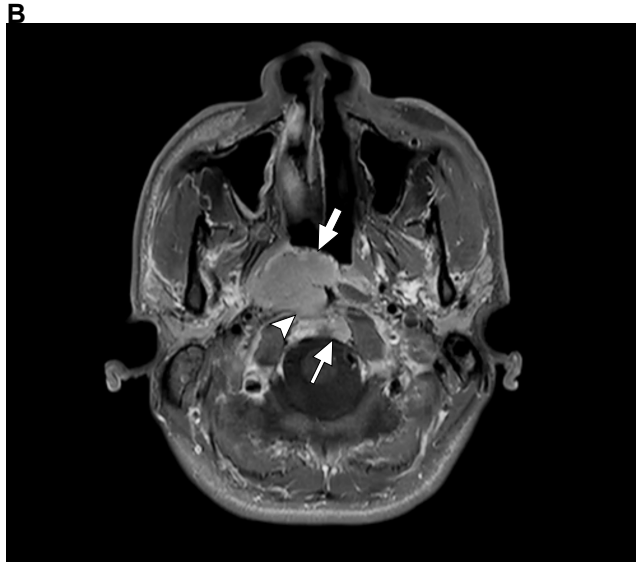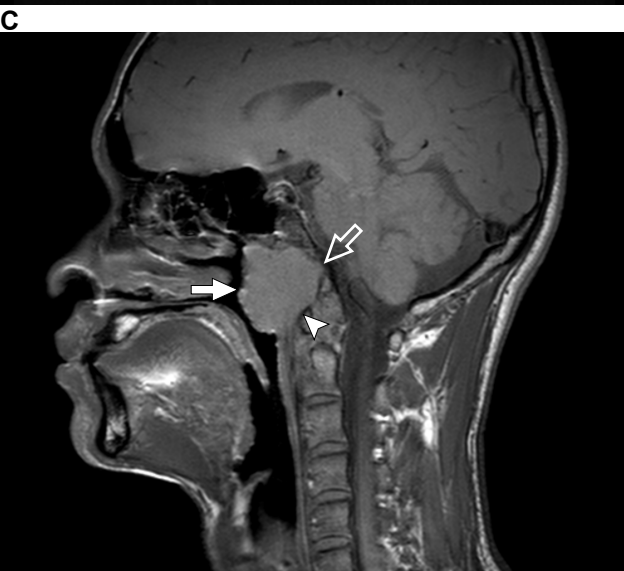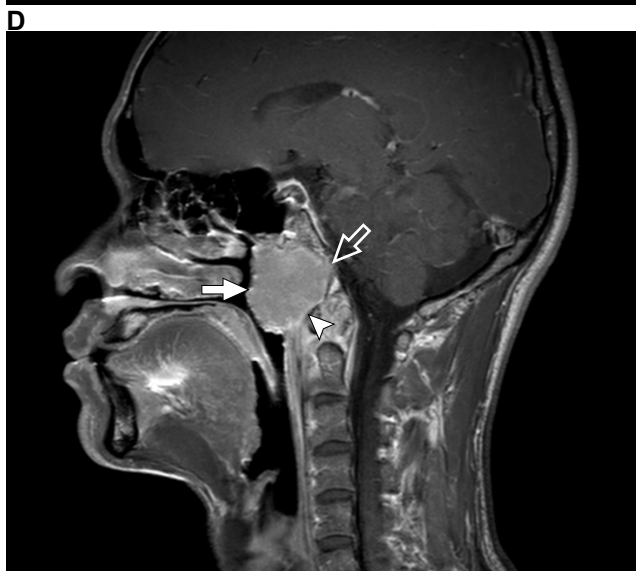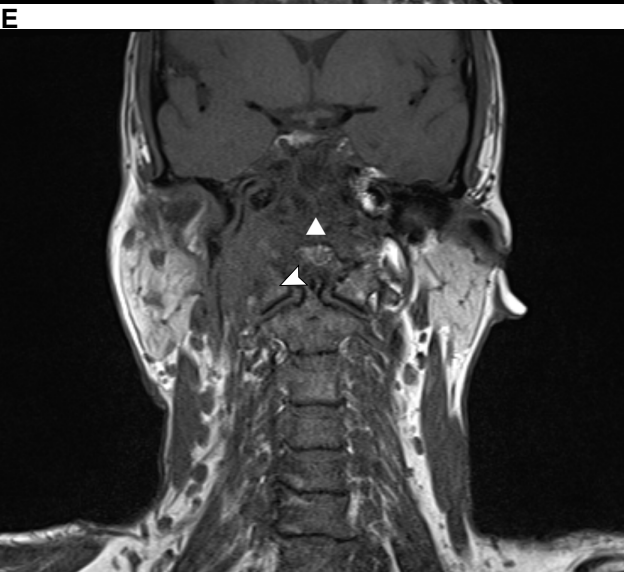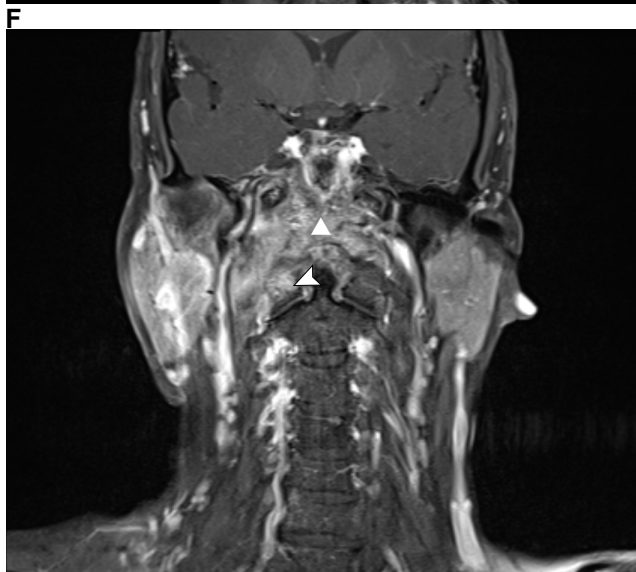

Supplement: Supplementary file 1 — Figure S1. The MR images of a 30‐year‐old man with invasion of cervical vertebrae presented in (A) axial T1‐weighted image, (B) axial T1‐weighted fat‐suppressed contrast‐enhanced image, (C) sagittal T1‐weighted image, (D) sagittal T1‐weighted contrast‐enhanced image, (E) coronal T1‐weighted image, and (F) coronal T1‐weighted fat‐suppressed contrast‐enhanced image: the mass of nasopharynx (solid thick arrow) posteriorly extended through the clivus (hollow thick arrow) and foramen magnum (solid triangle) and further invaded cervical vertebrae (solid arrowhead) and prepontine cistern (solid thin arrow). [file CTM2-10-e202-s001.pdf]

A

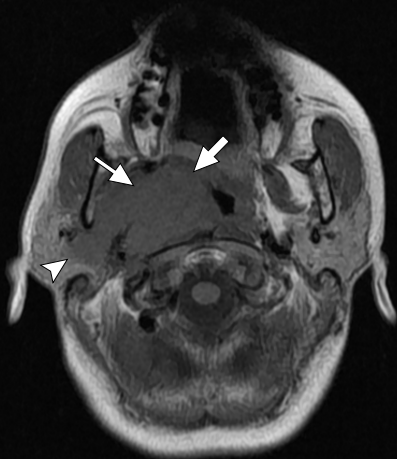

B

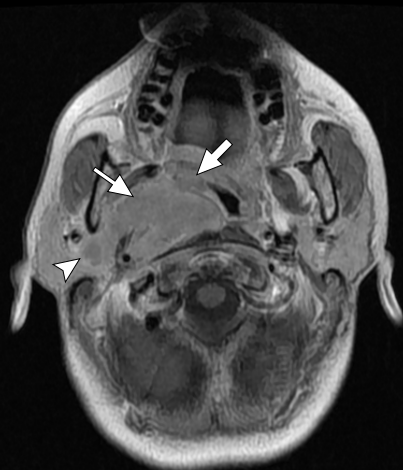

C

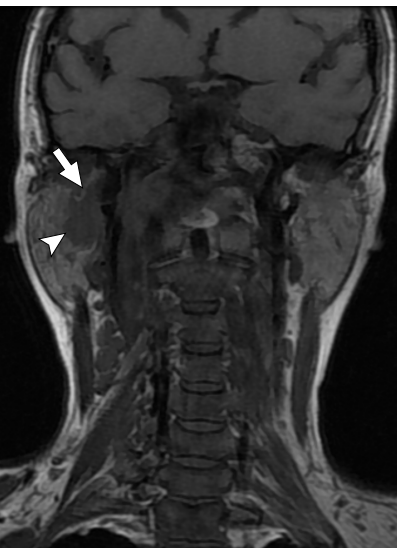

D

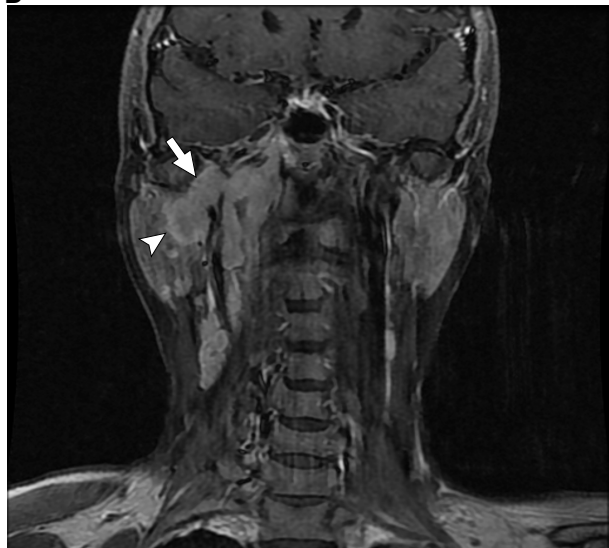

Supplement: Supplementary file 2 — Figure S2. The MR images of a 41‐year‐old woman with invasion of parotid gland presented in (A) axial T1‐weighted image, (B) axial T1‐weighted contrast‐enhanced image, (C) coronal T1‐weighted image, and (D) coronal T1‐weighted fat‐suppressed contrast‐enhanced image: the mass of nasopharynx (thick arrow) posterolaterally extended through the parapharyngeal fat space and adjacent soft tissue (thin arrow), and further invaded the deep lobe of parotid gland (arrowhead), resulting in a heterogeneous mass. [file CTM2-10-e202-s002.pdf]

**A**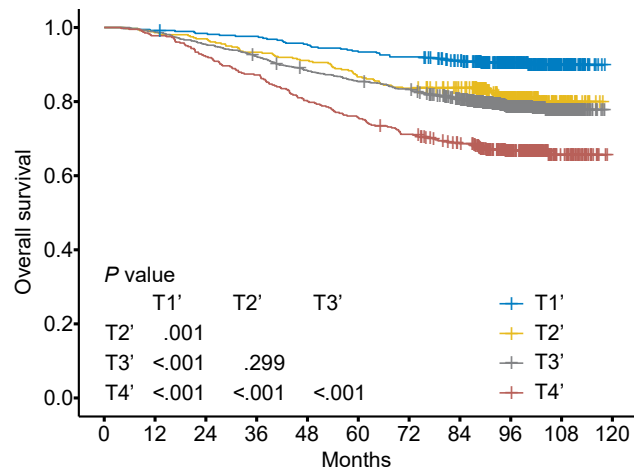

Number at risk

|     |      |      |     |     |     |     |     |     |     |     |   |
|-----|------|------|-----|-----|-----|-----|-----|-----|-----|-----|---|
| T1' | 379  | 376  | 372 | 369 | 360 | 353 | 348 | 329 | 193 | 51  | 0 |
| T2' | 360  | 355  | 349 | 336 | 328 | 312 | 301 | 288 | 163 | 35  | 0 |
| T3' | 1045 | 1034 | 997 | 962 | 921 | 890 | 867 | 805 | 487 | 107 | 0 |
| T4' | 406  | 397  | 374 | 354 | 325 | 307 | 288 | 266 | 160 | 42  | 0 |

**B**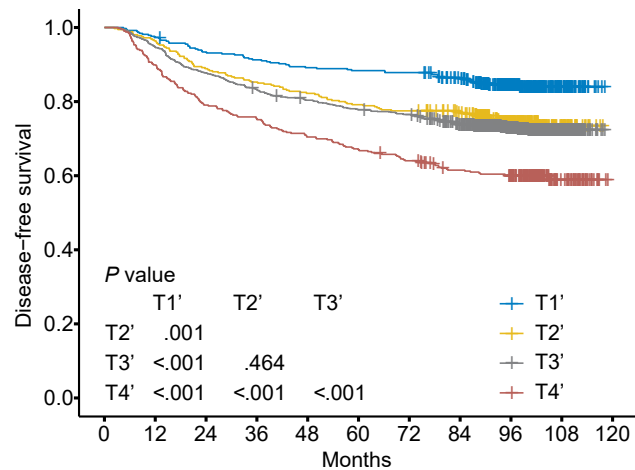

Number at risk

|     |      |     |     |     |     |     |     |     |     |    |   |
|-----|------|-----|-----|-----|-----|-----|-----|-----|-----|----|---|
| T1' | 379  | 369 | 353 | 345 | 337 | 334 | 332 | 311 | 179 | 48 | 0 |
| T2' | 360  | 347 | 320 | 307 | 296 | 285 | 279 | 264 | 150 | 33 | 0 |
| T3' | 1045 | 990 | 917 | 869 | 837 | 810 | 796 | 733 | 445 | 97 | 0 |
| T4' | 406  | 365 | 321 | 306 | 286 | 272 | 259 | 237 | 143 | 39 | 0 |

**C**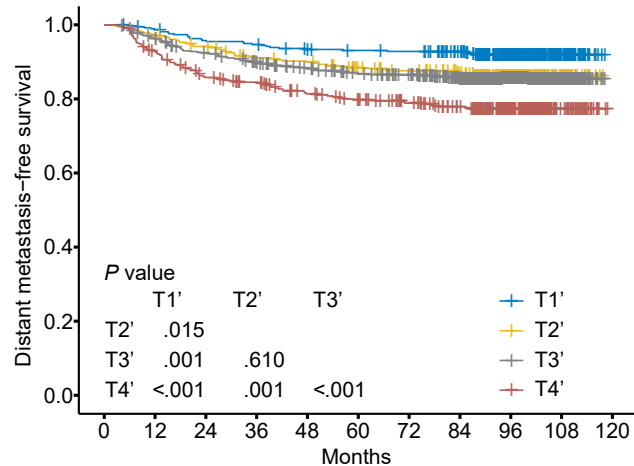

Number at risk

|     |      |      |     |     |     |     |     |     |     |     |   |
|-----|------|------|-----|-----|-----|-----|-----|-----|-----|-----|---|
| T1' | 379  | 372  | 360 | 356 | 347 | 344 | 342 | 325 | 189 | 51  | 0 |
| T2' | 360  | 350  | 337 | 321 | 316 | 301 | 292 | 277 | 158 | 34  | 0 |
| T3' | 1045 | 1002 | 953 | 918 | 888 | 859 | 841 | 779 | 473 | 105 | 0 |
| T4' | 406  | 372  | 339 | 326 | 302 | 288 | 270 | 249 | 149 | 39  | 0 |

**D**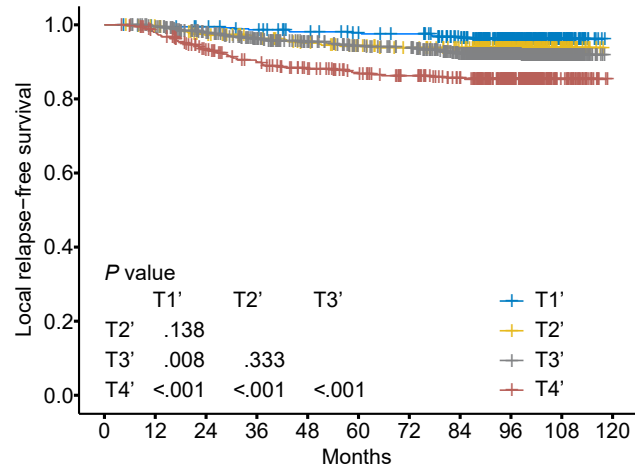

Number at risk

|     |      |      |     |     |     |     |     |     |     |     |   |
|-----|------|------|-----|-----|-----|-----|-----|-----|-----|-----|---|
| T1' | 379  | 375  | 370 | 364 | 355 | 348 | 345 | 323 | 188 | 48  | 0 |
| T2' | 360  | 355  | 340 | 327 | 315 | 304 | 295 | 282 | 160 | 35  | 0 |
| T3' | 1045 | 1028 | 977 | 933 | 894 | 859 | 844 | 776 | 471 | 104 | 0 |
| T4' | 406  | 391  | 355 | 333 | 311 | 294 | 279 | 257 | 154 | 41  | 0 |

Supplement: Supplementary file 3 — Figure S3. Kaplan‐Meier curves of overall survival (A), disease‐free survival (B), distant metastasis‐free survival (C), and local relapse‐free survival (D) according to the T’ category (in which cervical vertebrae invasion and parotid gland invasion were excluded from the frame of the 8th edition T category) for the whole cohort. [file CTM2-10-e202-s003.pdf]
