# Supplementary material for: New parameters of the 8th edition AJCC/UICC T category in nasopharyngeal carcinoma: Cervical vertebrae invasion and parotid gland invasion
Source: Clin Transl Med. 2020 Nov 10;10(7):e202. doi: 10.1002/ctm2.202 (PMC7654628; doi:10.1002/ctm2.202)
Supplement: Supplementary file 4 — Tables [file CTM2-10-e202-s004.docx]

| **Table S1. Patient characteristics categorized by cervical vertebrae invasion and parotid gland invasion** | | | | | | | |
| --- | --- | --- | --- | --- | --- | --- | --- |
| Characteristics | No. of patients (%) | | | | | | Total (n=2190) |
|  | With CVI | Without CVI | *P*-value | With PGI | Without PGI | *P*-value |  |
| Age (y) |  |  | .033 |  |  | .374 |  |
| ≤45 | 31 (39.2) | 1086 (51.4) |  | 20 (44.4) | 1097 (51.1) |  | 1117 (51.0) |
| >45 | 48 (60.8) | 1025 (48.6) |  | 25 (55.6) | 1048 (48.9) |  | 1073 (49.0) |
| Gender |  |  | .017 |  |  | .581 |  |
| Male | 68 (86.1) | 1567 (74.2) |  | 32 (71.1) | 1603 (74.7) |  | 1635 (74.7) |
| Female | 11 (13.9) | 544 (25.8) |  | 13 (28.9) | 542 (25.3) |  | 555 (25.3) |
| WHO histology |  |  | .357 |  |  | .221 |  |
| Type I | 1 (1.3) | 11 (0.5) |  | 1 (2.2) | 11 (0.5) |  | 12 (0.5) |
| Type II/III | 78 (98.7) | 2100 (99.5) |  | 44 (97.8) | 2134 (99.5) |  | 2178 (99.5) |
| T’ category^†^ |  |  | <.001 |  |  | <.001 |  |
| T1’ | 0 (0) | 379 (18.0) |  | 0 (0) | 379 (17.7) |  | 379 (17.3) |
| T2’ | 0 (0) | 360 (17.1) |  | 1 (2.2) | 359 (16.7) |  | 360 (16.4) |
| T3’ | 31 (39.2) | 1014 (48.0) |  | 17 (37.8) | 1028 (47.9) |  | 1045 (47.7) |
| T4’ | 48 (60.8) | 358 (17.0) |  | 27 (60.0) | 379 (17.7) |  | 406 (18.5) |
| N category^‡^ |  |  | .002 |  |  | .069 |  |
| N0 | 3 (3.8) | 363 (17.2) |  | 4 (8.9) | 362 (16.9) |  | 366 (16.7) |
| N1 | 44 (55.7) | 1211 (57.4) |  | 22 (48.9) | 1233 (57.5) |  | 1255 (57.3) |
| N2 | 19 (24.1) | 330 (15.6) |  | 11 (24.4) | 338 (15.8) |  | 349 (15.9) |
| N3 | 13 (16.5) | 207 (9.8) |  | 8 (17.8) | 212 (9.9) |  | 220 (10.0) |
| Clinical stage^†^ |  |  | <.001 |  |  | <.001 |  |
| I’ | 0 (0) | 128 (6.1) |  | 0 (0) | 128 (6.0) |  | 128 (5.8) |
| II’ | 0 (0) | 445 (21.1) |  | 1 (2.2) | 444 (20.7) |  | 445 (20.3) |
| III’ | 25 (31.6) | 1001 (47.4) |  | 15 (33.3) | 1011 (47.1) |  | 1026 (46.8) |
| IVA’ | 54 (68.4) | 537 (25.4) |  | 29 (64.4) | 562 (26.2) |  | 591 (27.0) |
| IC |  |  | <.001 |  |  | <.001 |  |
| Yes | 57 (72.2) | 1038 (49.2) |  | 37 (82.2) | 1058 (49.3) |  | 1095 (50.0) |
| No | 22 (27.8) | 1073 (50.8) |  | 8 (17.8) | 1087 (50.7) |  | 1095 (50.0) |
| CC |  |  | .367 |  |  | .113 |  |
| Yes | 64 (81.0) | 1618 (76.6) |  | 39 (86.7) | 1643 (76.6) |  | 1682 (76.8) |
| No | 15 (19.0) | 493 (23.4) |  | 6 (13.3) | 502 (23.4) |  | 508 (23.2) |
| Total | 79 (100.0) | 2111 (100.0) |  | 45 (100.0) | 2145 (100.0) |  | 2190 (100.0) |
| Abbreviations: CC = concurrent chemotherapy; CVI = cervical vertebrae invasion; IC = induction chemotherapy; PGI = parotid gland invasion; WHO = World Health Organization.  ^†^Cervical vertebrae invasion and parotid gland invasion were excluded from the frame of the 8^th^ edition T category.  ^‡^According to the 8^th^ edition of the American Joint Committee on Cancer/Union for International Cancer Control cancer staging manual. | | | | | | | |

| **Table S2. Association of primary disease extension and cervical vertebrae/parotid gland invasion** | | | | | | | | |
| --- | --- | --- | --- | --- | --- | --- | --- | --- |
| Primary disease extension | No. of patients (%) | | | | | | | |
|  | With CVI  (n=79) | Without CVI  (n=2111) | Unadjusted^*^ *P*-value | Adjusted^**^ *P*-value | With PGI  (n=45) | Without PGI  (n=2145) | Unadjusted^*^ *P*-value | Adjusted^**^ *P*-value |
| **Nasal cavity** | 0 (0) | 81 (3.8) | .141 | - | 0 (0) | 81 (3.8) | .353 | - |
| **Oropharynx** | 34 (43.0) | 129 (6.1) | <.001 | .003 | 28 (62.2) | 135 (6.3) | <.001 | <.001 |
| **Parapharyngeal space** | 79 (100.0) | 1732 (82.0) | <.001 | - | 45 (100.0) | 1766 (82.3) | .002 | - |
| **Adjacent soft tissue** | 78 (98.7) | 874 (41.1) |  |  | 44 (97.8) | 908 (42.3) |  |  |
| Medial pterygoid muscle | 65 (82.3) | 434 (20.6) | <.001 | - | 43 (95.6) | 456 (21.3) | <.001 | .002 |
| Lateral pterygoid muscle | 30 (38.0) | 139 (6.6) | <.001 | - | 28 (62.2) | 141 (6.6) | <.001 | .002 |
| Prevertebral muscle | 78 (98.7) | 793 (37.6) | <.001 | .099 | 43 (95.6) | 828 (38.6) | <.001 | - |
| **Skull base and Paranasal sinus** | 79 (100.0) | 1369 (64.9) |  |  | 44 (97.8) | 1404 (65.5) |  |  |
| Pterygoid process | 69 (87.3) | 1036 (49.1) | <.001 | - | 40 (88.9) | 1065 (49.7) | <.001 | - |
| Basis of sphenoid bone | 76 (96.2) | 1207 (57.2) | <.001 | - | 42 (93.3) | 1241 (57.9) | <.001 | - |
| Clivus | 72 (91.1) | 811 (38.4) | <.001 | - | 37 (82.2) | 846 (39.4) | <.001 | .030 |
| Petrous apex | 75 (94.9) | 768 (36.4) | <.001 | - | 42 (93.3) | 801 (37.3) | <.001 | - |
| Great wing of sphenoid bone | 44 (55.7) | 307 (14.5) | <.001 | - | 27 (60.0) | 324 (15.1) | <.001 | - |
| Foramen magnum | 77 (97.5) | 73 (3.5) | <.001 | <.001 | 37 (82.2) | 113 (5.3) | <.001 | <.001 |
| Skull base foramina^†^ | 74 (93.7) | 589 (27.9) | <.001 | - | 41 (91.1) | 622 (29.0) | <.001 | - |
| Paranasal sinus | 31 (39.2) | 304 (14.4) | <.001 | - | 17 (37.8) | 318 (14.8) | <.001 | - |
| **T4 parameters** | 43 (54.4) | 336 (15.9) |  |  | 26 (57.8) | 353 (16.5) |  |  |
| Intracranial | 40 (50.6) | 304 (14.4) | <.001 | - | 23 (51.1) | 321 (15.0) | <.001 | - |
| Hypopharynx | 0 (0) | 10 (0.5) | >.999 | .998 | 1 (2.2) | 9 (0.4) | .188 | - |
| Orbit | 12 (15.2) | 53 (2.5) | <.001 | - | 6 (13.3) | 59 (2.8) | <.001 | - |
| Extensive soft tissue | 9 (11.4) | 63 (3.0) | <.001 | - | 6 (13.3) | 66 (3.1) | .001 | - |
| Abbreviations: CVI = cervical vertebrae invasion; PGI = parotid gland invasion.  ^*^Unadjusted *P*-value, Chi-square test.  ^**^Adjusted *P*-value, logistic regression.  ^†^Skull base foramina included foramen rotundum, foramen ovale, foramen spinosum, jugular foramen, foramen lacerum, pterygopalatine fossa, and hypoglossal canal. | | | | | | | | |

| **Table S3. Dosimetric data of selected organs at risk and target volumes for patients with cervical vertebrae/parotid gland invasion** | |
| --- | --- |
|  | Mean (range) |
| Brainstem PRV |  |
| D_max_ (Gy) | 68.0 (56.3-80.0) |
| D_1_ (Gy) | 63.6 (51.7-74.3) |
| D_0.03cc_ | 66.1 (54.9-77.5) |
| Spinal cord PRV |  |
| D_max_ (Gy) | 50.2 (39.4-65.4) |
| D_1_ (Gy) | 42.7 (36.3-55.1) |
| D_0.03cc_ | 46.4 (37.5-61.2) |
| Left temporal lobe |  |
| D_max_ (Gy) | 72.3 (42.5-83.6) |
| D_1_ (Gy) | 65.4 (32.8-77.3) |
| D_0.03cc_ | 69.8 (40.7-81.2) |
| Right temporal lobe |  |
| D_max_ (Gy) | 73.0 (53.7-80.4) |
| D_1_ (Gy) | 66.8 (38.9-76.6) |
| D_0.03cc_ | 71.6 (49.8-78.8) |
| Left parotid gland |  |
| D_mean_ (Gy) | 42.2 (31.5-64.5) |
| D_50_ (Gy) | 39.7 (27.2-66.9) |
| Right parotid gland |  |
| D_mean_ (Gy) | 43.9 (31.3-69.8) |
| D_50_ (Gy) | 41.7 (27.0-70.7) |
| GTV-P |  |
| Volume (mL) | 95.8 (9.8-246.6) |
| D_mean_ (Gy) | 73.8 (68.5-76.5) |
| D_min_ (Gy) | 65.2 (41.6-72.4) |
| D_99_ (Gy) | 70.0 (60.7-74.3) |
| D_95_ (Gy) | 71.4 (66.5-74.6) |
| D_1_ (Gy) | 77.0 (71.8-79.8) |
| V_100_ (%) | 97.8 (45.7-100.0) |
| V_95_ (%) | 99.8 (96.0-100.0) |
| PTV-GTV-P |  |
| D_mean_ (Gy) | 73.3 (68.1-76.3) |
| D_min_ (Gy) | 58.2 (29.8-69.5) |
| D_99_ (Gy) | 68.0 (56.5-73.1) |
| D_95_ (Gy) | 70.3 (64.1-74.1) |
| D_1_ (Gy) | 76.9 (71.6-79.7) |
| V_100_ (%) | 94.9 (39.4-100.0) |
| V_95_ (%) | 99.2 (91.7-100.0) |
| Abbreviations: D_max_ = maximum point dose; D_mean_ = mean dose; D_min_ = minimum point dose; D_1_ = maximum dose to 1% volume; D_50_ = minimum dose to 50% volume; D_95_ = minimum dose to 95% volume; D_99_ = minimum dose to 99% volume; D_0.03cc_ = maximum dose to 0.03 cm^3^ volume; GTV-P = Primary gross tumor volume; PRV = Planning risk volume; PTV-GTV-P = Planning target volume of primary gross tumor volume;V_100_ = percentage volume receiving at least 100% of prescribed dose; V_95_ = percentage volume receiving at least 95% of prescribed dose. | |

| **Table S4. Multivariable Cox regression analysis for OS, DFS, DMFS, and LRFS in the whole cohort** | | | | | | | | | | | |
| --- | --- | --- | --- | --- | --- | --- | --- | --- | --- | --- | --- |
| Variable | OS | |  | DFS | |  | DMFS | |  | LRFS | |
|  | HR (95%CI) | *P*-value^*^ |  | HR (95%CI) | *P*-value^*^ |  | HR (95%CI) | *P*-value^*^ |  | HR (95%CI) | *P*-value^*^ |
| Age (y) |  |  |  |  |  |  |  |  |  |  |  |
| ≤45 | 1 | - |  | 1 | - |  | - | - |  | 1 | - |
| >45 | 1.57 (1.31-1.90) | <.001 |  | 1.44 (1.23-1.70) | <.001 |  | - | - |  | 1.51 (1.11-2.07) | .009 |
| Gender |  |  |  |  |  |  |  |  |  |  |  |
| Male | 1 | - |  | 1 | - |  | 1 | - |  | - | - |
| Female | 0.79 (0.63-0.99) | .038 |  | 0.83 (0.68-1.01) | .057 |  | 0.68 (0.51-0.91) | .009 |  | - | - |
| WHO histology |  |  |  |  |  |  |  |  |  |  |  |
| Type I | - | - |  | - | - |  | - | - |  | 1 | - |
| Type II/III | - | - |  | - | - |  | - | - |  | 0.25 (0.08-0.79) | .018 |
| T’ category^†^ |  |  |  |  |  |  |  |  |  |  |  |
| T1’ | 1 | - |  | 1 | - |  | 1 | - |  | 1 | - |
| T2’ | 1.62 (1.08-2.42) | .020 |  | 1.42 (1.02-1.97) | .039 |  | 1.39 (0.88-2.20) | .162 |  | 1.62 (0.82-3.19) | .161 |
| T3’ | 1.88 (1.32-2.68) | <.001 |  | 1.55 (1.16-2.06) | .003 |  | 1.51 (1.02-2.25) | .041 |  | 2.07 (1.17-3.66) | .012 |
| T4’ | 3.00 (2.06-4.35) | <.001 |  | 2.40 (1.77-3.26) | <.001 |  | 2.38 (1.56-3.66) | <.001 |  | 4.05 (2.25-7.30) | <.001 |
| N category^‡^ |  |  |  |  |  |  |  |  |  |  |  |
| N0 | 1 | - |  | 1 | - |  | 1 | - |  | - | - |
| N1 | 1.55 (1.09-2.19) | .014 |  | 1.59 (1.19-2.14) | .002 |  | 1.53 (0.99-2.37) | .058 |  | - | - |
| N2 | 2.63 (1.80-3.83) | <.001 |  | 2.66 (1.93-3.69) | <.001 |  | 3.17 (1.99-5.06) | <.001 |  | - | - |
| N3 | 5.07 (3.47-7.40) | <.001 |  | 4.36 (3.13-6.06) | <.001 |  | 6.05 (3.80-9.62) | <.001 |  | - | - |
| CVI |  |  |  |  |  |  |  |  |  |  |  |
| No | 1 | - |  | 1 | - |  | 1 | - |  | - | - |
| Yes | 1.65 (1.16-2.36) | .006 |  | 1.41 (1.01-1.98) | .046 |  | 1.66 (1.08-2.54) | .020 |  | - | - |
| Abbreviations: CI = confidence interval; CVI = cervical vertebrae invasion; DFS = disease-free survival; DMFS = distant metastasis-free survival; HR = hazard ratio; LRFS = local relapse-free survival; OS = overall survival; WHO = World Health Organization. | | | | | | | | | | | |
| ^*^Multivariable *P*-values were calculated using an adjusted Cox proportional hazards model. The following parameters were included in the Cox proportional hazards model: age (>45 vs ≤45), gender (female vs male), WHO histology (type II/III vs type I), induction chemotherapy (yes vs no), concurrent chemotherapy (yes vs no), T’ category, N category, cervical vertebrae invasion (yes vs no), and parotid gland invasion (yes vs no).  ^†^Cervical vertebrae invasion and parotid gland invasion were excluded from the frame of the 8^th^ edition T category.  ^‡^According to the 8^th^ edition of the American Joint Committee on Cancer/Union for International Cancer Control cancer staging manual. | | | | | | | | | | | |

| **Table S5. Performance evaluation of the 8^th^ edition T category and proposed T category based on overall survival in the validation set** | | |
| --- | --- | --- |
| Evaluation criteria | 8^th^ edition T category | Proposed T category |
| % Hazard consistency | 1.628 | 0.652 |
| Score | 0.632 | 0.369 |
| Rank | 1.632 | 1.369 |
| Hazard discrimination | 0.794 | 0.523 |
| Score | 0.747 | 0.254 |
| Rank | 1.747 | 1.254 |
| Balance | 0.448 | 0.399 |
| Score | 0.839 | 0.158 |
| Rank | 1.839 | 1.158 |
| Outcome prediction (% variance explained) | 24.8 | 24.9 |
| Score | 0.520 | 0.480 |
| Rank | 1.520 | 1.480 |
| Overall score | 2.737 | 1.260 |
| Overall rank | 1.625 | 1.094 |
| % Rank = 1 | 751 | 1812 |

**Figure legends**

**Figure S1.** The MR images of a 30-year-old man with invasion of cervical vertebrae presented in (**A**) axial T1-weighted image, (**B**) axial T1-weighted fat-suppressed contrast-enhanced image, (**C**) sagittal T1-weighted image, (**D**) sagittal T1-weighted contrast-enhanced image, (**E**) coronal T1-weighted image, and (**F**) coronal T1-weighted fat-suppressed contrast-enhanced image: the mass of nasopharynx (solid thick arrow) posteriorly extended through the clivus (hollow thick arrow) and foramen magnum (solid triangle) and further invaded cervical vertebrae (solid arrowhead) and prepontine cistern (solid thin arrow).

**Figure S2.** The MR images of a 41-year-old woman with invasion of parotid gland presented in (**A**) axial T1-weighted image, (**B**) axial T1-weighted contrast-enhanced image, (**C**) coronal T1-weighted image, and (**D**) coronal T1-weighted fat-suppressed contrast-enhanced image: the mass of nasopharynx (thick arrow) posterolaterally extended through the parapharyngeal fat space and adjacent soft tissue(thin arrow), and further invaded the deep lobe of parotid gland (arrowhead), resulting in a heterogeneous mass.

**Figure S3.** Kaplan-Meier curves of overall survival (**A**), disease-free survival (**B)**, distant metastasis-free survival (**C)**, and local relapse-free survival (**D**) according to the T’ category (in which cervical vertebrae invasion and parotid gland invasion were excluded from the frame of the 8^th^ edition T category) for the whole cohort.
